# A prospective study on the effects of 10 whole-body cryotherapy sessions on skin parameters in young healthy women

**DOI:** 10.3389/fphys.2026.1812461

**Published:** 2026-06-05

**Authors:** Bartłomiej Ptaszek, Magdalena Uzar, Szymon Podsiadło, Janka Poráčová, Olga Czerwińska-Ledwig

**Affiliations:** 1 Institute of Applied Sciences, University of Physical Culture in Krakow, Krakow, Poland; 2 Faculty of Motor Rehabilitation, University of Physical Culture in Krakow, Krakow, Poland; 3 Institute of Clinical Rehabilitation, University of Physical Culture in Krakow, Krakow, Poland; 4 Faculty of Humanities and Natural Sciences, University of Prešov, Presov, Slovakia; 5 Institute of Basic Sciences, University of Physical Culture in Krakow, Krakow, Poland

**Keywords:** skin, skin elasticity, skin firmness, skin hydration, TEWL, whole-body cryotherapy

## Abstract

**Background/objectives:**

Whole-body cryotherapy (WBC) uses extremely low temperatures to induce physiological responses, directly affecting skin perfusion and metabolism. The aim of the study was to evaluate the effect of a series of 10 whole-body cryotherapy sessions on selected skin parameters in 12 young, healthy, and non-training women. Methods: Participants aged 21–25 underwent a series of 10 cryotherapy sessions at a temperature of -120 °C. Measurements were performed at 7 time points using the following probes (Courage Khazaka, Germany): Corneometer (skin hydration), Tewameter (transepidermal water loss, TEWL), Cutometer^®^ dual MPA 580 (skin elasticity and firmness), Mexameter (erythrema and melanin content), and Skin-Thermometer^®^ ST 500 (skin temperature).

**Results:**

The study revealed a statistically significant decrease in skin temperature and hydration, as well as an increase in redness (erythema). No significant changes were observed in biomechanical parameters (firmness, elasticity) or in TEWL.

**Conclusions:**

Whole-body cryotherapy in young, healthy women induces a marked vascular response, affecting skin microcirculation. Despite the reduction in hydration, the treatments are likely safe for the skin’s protective barrier and do not negatively impact its mechanical properties in young, healthy women. Further research is needed to explain the complexity of these processes.

## 1 Introduction

Cryotherapy is one of the oldest fields of physiotherapy. It encompasses a wide range of activities, from pain-relieving treatments to those supporting biological regeneration in sports and aesthetic medicine. Extremely low temperatures affect the circulatory and endocrine systems (Korzonek-Szlacheta et al., 2007) and have metabolic effects (Lubkowska et al., 2010). WBC treatments improve tissue blood flow, increase their nourishment, and support the removal of toxins from the body (Stanek and Sieroń, 2012) while also improving quality of life (Piotrowska et al., 2021). It is also believed to have a beneficial effect on the characteristics of our skin, such as hydration, redness and elasticity. Human skin is considered one of the largest organs in the body. It consists of three layers: the epidermis, dermis, and subcutaneous tissue (Kieć-Świerczyńska and Kręcisz, 2011). The stratum corneum serves as a key protective barrier and is responsible for water retention in the body (water homeostasis). The dermis is richly vascularized and innervated; blood vessels transport nutrients through diffusion, and nerve endings enable constant contact with the external environment. The physiological response to cold depends on both the applied temperature and the extent of the exposed body surface (Heider and Jastrząb, 2013; Wolski and Kędzia, 2019). Initially, vasoconstriction occurs, significantly reducing–heat transport to peripheral tissues (Botonis and Kounalakis, 2017). This process initiates a reaction that leads to a decrease in skin temperature and reduced blood flow through the skin (Hohenauer et al., 2019). Subsequently, blood vessels dilate, leading to a vascular response, i.e., tissue hyperemia (Cichoń et al., 2010). Active reperfusion of cutaneous microcirculation persists for up to several hours after a single systemic cryotherapy treatment, and a series of treatments leads to increased cutaneous microcirculation reactivity (Szczyguła et al., 2011).

The stratum corneum creates a tight barrier between the moist internal environment and the dry external environment. At the same time, constant passive diffusion towards the surface, i.e., TEWL, occurs (Akdeniz et al., 2018). Proper tissue hydration depends on both the transport of water from the deeper layers of the dermis and the tightness of the epidermal barrier (Wolski and Kędzia, 2019). The structural basis for the mechanical resistance and appearance of skin is the biomechanical properties of the dermis, referred to as viscoelasticity. Viscoelasticity is also an important indicator of skin function, enabling the study of its physiological functions from a mechanical perspective (Liu et al., 2015). With age, the levels of elastin, collagen, and hyaluronic acid gradually decline, contributing to the loss of structural integrity and elasticity (Baumann et al., 2021). Most studies on WBC focus on patient populations, and few assess the impact on young, healthy individuals, which is why we decided to focus our study on this group. Skin nourishment is determined by cutaneous microcirculation, which is characterized by high dynamics (Konturek, 2013). Due to the close physiological interdependence of the described mechanisms, only simultaneous analysis of all these parameters allows for a reliable assessment of the functional state of the skin.

## 2 Materials and methods

### 2.1 Participant characteristics

The presented prospective study, consistent with the assumptions of the Helsinki Declaration; approval number of the Bioethical Committee of the District Medical Chamber in Krakow: 64/KBL/OIL/2024 of 04/07/2024; each volunteer read the information about the study design and was given the opportunity to ask questions, after which he gave informed written consent to participate in the study; A physiotherapist took care of the participants’ safety.

The study involved 12 (n=12) women aged 21–25 years (mean age: 22.58 ± 1.68 years). The participants had an average BMI of 20.83, with a height of 158–182 cm (mean height: 168.00 ± 0.07 cm). The study lasted a total of four weeks, during which four sessions of measurement of selected skin parameters were conducted. Measurements took place at the Academy of Physical Culture in Krakow and at the Małopolska Cryotherapy Center in Krakow. To ensure homogeneity within the study group, the participants did not regularly engage in high-intensity physical activity (professional training). The participants were healthy (without chronic diseases) and were required to undergo medical examinations, which also ruled out contraindications to WBC treatments. Main exclusion criteria: chronic diseases, infections during the study period, epidermal rupture, pregnancy, elevated temperature, cardiovascular diseases, coagulation disorders, hypersensitivity to cold, metal implants, use of stimulants, change in physical activity immediately before and during the project, change in diet immediately before and during the project.

All participants formed a cohesive group of women who led a moderately active lifestyle, engaged in similarly intense occupational activities, and were not sedentary. The participants followed a healthy, balanced diet (Mediterranean or DASH (Dietary Approaches to Stop Hypertension)). None of the women had previously undergone whole-body or local cryotherapy treatments. All of these guidelines allowed for the achievement of results suggesting an independent intervention, unaffected by external factors such as physical activity or health status.

### 2.2 Measuring tools

Skin parameters assessment was performed seven times within a strictly defined timeframe, aiming to capture immediate and long-term changes. The first measurements were taken seven days before the start of the WBC treatment (control), which served to establish a baseline skin condition. Then, on the first day of the treatment series, measurements were taken twice: before entering the cryochamber and 5 minutes after exiting the chamber. On the last day (day 10) of the treatment series, measurements were taken three times: before entering the cryochamber, 5 minutes after exiting the chamber, and 15 minutes after exiting. The final (follow-up) measurement was performed seven days after the last WBC treatment to assess the durability and long-term effects of the changes in skin properties.

To determine skin parameters and evaluate selected aspects of the participants skin condition, measurement equipment was used (Courage Khazaka, Germany). Each measurement was performed three times in on volar forearm skin surface and then average value was calculated. The study analyzed five key parameters: skin surface hydration (Corneometer^®^ CM 825), transepidermal water loss (Tewameter^®^ TM 300), skin elasticity and coefficient of friction (Cutometer^®^ dual MPA 580), temperature (Skin-Thermometer ST 500), melanin level and redness (Mexameter^®^ MX 18).

All measurements were conducted in a single, specially prepared room to ensure controlled environmental conditions and avoid factors that could influence the results. A constant temperature of 20–24 degrees Celsius and humidity of 30-50% were maintained throughout the measurements. Furthermore, measurements were taken at similar times of day, minimizing the impact of circadian rhythms on skin condition.

### 2.3 Description of the intervention

Each WBC treatment was conducted in two stages. Participants initially spent approximately 20–30 seconds in a vestibule with a temperature of -60 degrees Celsius, then moved to a target chamber, where they remained for three minutes at -120 degrees Celsius. During exposure to low temperatures, participants were required to wear protective equipment, including gloves, a hat, a mask covering their mouth and nose, long cotton or wool socks, and wooden shoes. Treatments were performed daily from Monday to Friday at a fixed time (3:00-4:00 PM) for two weeks.

### 2.4 Statistical analysis

The empirical analysis allowed for the presentation of descriptive statistics for the studied parameter in the form of means, standard deviations, and minimum and maximum values. The normality of the distribution of variables was verified using the Shapiro-Wilk test. The ANOVA/MANOVA test for repeated measures was used to determine the significance of changes within the study group depending on the time of measurement. ANOVA is considered a univariate method because it examines the effect of one or more independent variables on a single dependent variable. In contrast, MANOVA is a multivariate method. It analyzes the effect of independent variables on multiple dependent variables simultaneously. in summary, ANOVA focuses on one outcome variable, whereas MANOVA examines multiple outcome variables within a single analysis, providing a broader view of how groups differ across several dimensions. In the case of statistically significant differences in the analysis of variance, *post-hoc* tests (The Tukey HSD test was used as a *post hoc* analysis following ANOVA to identify which group means differed significantly from each other) were performed. The significance level for the analyses was p=0.05. All calculations were performed using Statistica 13 software (Tibco Software Inc., USA).

## 3 Results

The average results of individual measurements are given in **Table 1**.

**Table 1:** Tested indicators (mean ± standard deviation) and analysis of variance for repeated measurements of the tested indicators – F test value and p significance level.

Parameters	I	II	III	IV	V	VI	VII	FF	P
Thermometer (∘C)	32.70 ± 0.75	32.70 ± 0.75	27.57 ± 2.79	30.34 ± 1.06	24.12 ± 4.27	29.51 ± 1.06	30.64 ± 1.23	25.35	p < 0.001
Corneometer	54.90 ± 7.65	54.9 ± 7.65	58.59 ± 7.16	43.10 ± 15.45	49.79 ± 16.44	43.41 ± 11.85	41.13 ± 11.98	9.27	p < 0.001
Mexameter (Erythema)	54.80 ± 19.64	54.80 ± 19.64	121.0 ± 64.27	135.50 ± 58.47	167.90 ± 86.89	136.40 ± 56.52	159.00 ± 45.05	14.95	p < 0.001
Tewameter (Avg.)	14.40 ± 4.73	14.40 ± 4.72	13.96 ± 5.88	11.62 ± 9.22	12.36 ± 7.43	10.08 ± 6.97	10.85 ± 2.51	1.83	0.10
Tewameter (Spread)	0.22 ± 0.20	0.22 ± 0.20	0.19 ± 0.14	0.11 ± 0.04	0.13 ± 0.08	0.11 ± 0.04	0.09 ± 0.02	2.86	0.02
Cutometer R0 (mm)	0.31 ± 0.06	0.31 ± 0.06	0.33 ± 0.06	0.29 ± 0.06	0.30 ± 0.06	0.30 ± 0.05	0.29 ± 0.03	1.92	0.09
Cutometer R1 (mm)	0.06 ± 0.01	0.06 ± 0.01	0.06 ± 0.01	0.06 ± 0.02	0.05 ± 0.01	0.05 ± 0.01	0.06 ± 0.01	1.29	0.26
Cutometer R2 (%)	80.9 ± 3.64	80.9 ± 3.64	84.84 ± 6.92	81.57 ± 8.11	83.56 ± 6.35	82.38 ± 5.58	79.93 ± 4.46	1.48	0.20
Cutometer R3 (mm)	0.31 ± 0.06	0.31 ± 0.06	0.33 ± 0.06	0.29 ± 0.06	0.30 ± 0.06	0.30 ± 0.05	0.29 ± 0.03	1.92	0.09
Cutometer R4 (mm)	0.06 ± 0.01	0.06 ± 0.01	0.06 ± 0.01	0.06 ± 0.02	0.05 ± 0.01	0.05 ± 0.01	0.06 ± 0.01	1.29	0.28
Cutometer R5 (%)	87.60 ± 8.98	87.60 ± 8.98	91.40 ± 7.00	86.68 ± 8.98	89.67 ± 7.44	86.22 ± 7.15	85.36 ± 7.84	1.14	0.35

Statistical analysis revealed significant differences in the mean values of selected skin parameters, which were verified using repeated measures ANOVA (**Tables 1**, **2**). The analysis revealed significant:

**Table 2:** *Post hoc* test values for the tested indicators.

Parameter	Study	I	II	III	IV	V	VI	VII
Thermometer	I	–	1.00	0.00	0.01	0.00	0.00	0.02
	II	1.00	–	0.00	0.01	0.00	0.00	0.02
	III	0.00	0.00	–	0.00	0.00	0.03	0.00
	IV	0.01	0.01	0.00	–	0.00	0.33	0.73
	V	0.00	0.00	0.00	0.00	–	0.00	0.00
	VI	0.00	0.00	0.03	0.33	0.00	–	0.19
	VII	0.02	0.02	0.00	0.73	0.00	0.19	–
Corneometer	I	–	1.00	0.27	0.00	0.11	0.00	0.00
	II	1.00	–	0.27	0.00	0.11	0.00	0.00
	III	0.27	0.27	–	0.00	0.01	0.00	0.00
	IV	0.00	0.00	0.00	–	0.04	0.92	0.55
	V	0.11	0.11	0.01	0.04	–	0.05	0.01
	VI	0.00	0.00	0.00	0.92	0.05	–	0.48
	VII	0.00	0.00	0.00	0.55	0.01	0.48	–
Mexameter	I	–	1.00	0.00	0.00	0.00	0.00	0.00
	II	1.00	–	0.00	0.00	0.00	0.00	0.00
	III	0.00	0.00	–	0.39	0.01	0.37	0.03
	IV	0.00	0.00	0.39	–	0.06	0.96	0.17
	V	0.00	0.00	0.01	0.06	–	0.07	0.60
	VI	0.00	0.00	0.37	0.96	0.07	–	0.19
	VII	0.00	0.00	0.03	0.17	0.60	0.19	–
Tewametr	I	–	1.00	0.53	0.02	0.07	0.02	0.01
	II	1.00	–	0.53	0.02	0.07	0.02	0.01
	III	0.53	0.53	–	0.07	0.23	0.09	0.04
	IV	0.02	0.02	0.07	–	0.54	0.91	0.75
	V	0.07	0.07	0.23	0.54	–	0.62	0.36
	VI	0.02	0.02	0.09	0.91	0.62	–	0.67
	VII	0.01	0.01	0.04	0.75	0.36	0.67	–

- decrease in skin temperature measured with a thermometer, with the largest differences observed between study I and V, II and V. Significant decreases also occurred between study I and III, I and IV, I and VI, I and VII, and similarly between study II and III, IV, VI, VII, as well as between study III and V, IV, V (F = 25.35, p < 0.001) (An initial decrease in skin temperature immediately after treatment, followed by a reactive increase 15 minutes later);
- increase in skin temperature measured by thermometer, with the highest increase between study V and VI, and the remaining increases between study III and IV, III and VI, III and VII, V and VII (F = 25.35, p < 0.001);
- decrease in skin hydration measured with a corneometer, with the largest difference occurring between study III and VII. Furthermore, differences were observed between study I and IV, I and VI, I and VII, II and IV, II and VI, II and VII, III and IV, III and V, III and VI, V and VII (F = 9.27, p < 0.001);
- decrease in the transepidermal water loss (Tewametr Spread) between study I and VII, I and IV, I and VI, II and IV, II and VI, II and VII, III and VII (F = 2.86, p = 0.02);
- increase in the level of erythema measured by mexameter, with the highest increase observed between study I and V and II and V. Furthermore, statistically significant increases occurred between study I and III, I and IV, I and VI, I and VII, and similarly for study II in relation to III, IV, VI, VII, as well as between study III and V, III and VII (F = 14.95, p < 0.001);

In the case of the remaining parameters: transepidermal water loss measured with a tewameter (Tewameter Robust Avg.) and all parameters of skin elasticity and firmness measured with a cutometer (R0, R1, R2, R3, R4, R5) there were no statistically significant differences between subsequent measurements. Tewameter: (F = 1.83, p=0.11), Cutometer: R0: (F = 1.92, p=0.09), R1: (F = 1.29, p=0.28), R2: (F = 1.48, p=0.20), R3: (F = 1.92, p=0.09), R4: (F = 1.29, p=0.28) R5: (F = 1.14, p=0.35).

## 4 Discussion

The main aim of this study was to assess the effect of a series of 10 WBC treatments on selected skin characteristics in young, healthy, and non-competitively training women. This group constitutes an important contribution to previous studies, which largely focus on individuals with various medical conditions and illnesses (Dzidek and Piotrowska, 2022). The obtained results, in conjunction with the current available literature, allow for the formulation of important conclusions regarding both the therapeutic potential and limitations of cryotherapy in wellness treatments. This allows for the isolation of the physical effect of cold, unaffected by ongoing inflammatory processes or pharmacotherapy, which is difficult to capture in clinical patient groups.

The most pronounced effect observed in our study is the dynamic thermal response of the skin obtained from measurements taken after the WBC procedure. The observed statistically significant drop in superficial temperature on the forearm immediately after the treatment, followed by a subsequent increase after 15 minutes, is a manifestation of the thermoregulatory mechanism. Thermographic studies have demonstrated that vasoconstriction is the body’s first defensive reaction to heat loss. The magnitude of the skin’s thermal response to WBC depends on individual patient characteristics, such as BMI (Cholewka et al., 2012). Therefore, to standardize the results, the study group demonstrates high homogeneity. The need for such selection is confirmed by studies examining the effect of varying body mass index on skin parameters after WBC. It has been shown that the thickness of subcutaneous adipose tissue acts as a thermal insulator, which causes individuals with a higher BMI to demonstrate different skin response dynamics compared to individuals with a normal weight (Dzidek et al., 2025).

The increased skin redness (erythema) observed in the study is not merely a superficial thermal symptom. The hyperemia, visible as a change in skin color, indicates improved microcirculation, which is a sign of metabolic changes. In the second phase of the cold response, the warming phase, during which erythema can be observed, metabolic processes intensify in the treated tissues (Lubkowska, 2012). The results obtained in this study confirm the occurrence of the described physiological response, indicating a normal response of the vascular system of the studied women. The increased skin redness is also a clinical reflection of improved endothelial homeostasis and improved microcirculation via a biochemical pathway (reduced myeloperoxidase activity, which limits nitric oxide consumption, directly counteracting epithelial dysfunction) (Stanek et al., 2020).

The statistically significant decrease in skin hydration over the course of the treatment series indirectly contradicts some scientific reports suggesting a beneficial effect of cryotherapy on skin condition. In a patient with atopic dermatitis, an increase in skin hydration was demonstrated after a series of 15 WBC treatments. In this case study, the improvement in this parameter may have resulted from the anti-inflammatory effect of cryotherapy, which strengthened the already damaged skin barrier (Wysopal et al., 2021). In our study, the possible physical effect of dry gas predominated. Aditionally, a single treatment was also shown to have no significant effect on hydration levels. As the authors of before cited study suggest, a single treatment may be too weak a stimulus to induce significant changes in the structure of the epidermis (Wysopal et al., 2021). In the present study, cyclical exposure to cold led to cumulative effects, revealing changes invisible after a single exposure to the thermal agent. In work of Piotrowska et al. no effect of a single treatment skin hydration was shown (Piotrowska et al., 2021). Our results may suggest that repeated cold treatments should be combined with moisturizing care to compensate for the dry environment of the cryochamber.

Analysis of TEWL, an indicator of skin hydrolipid barrier function, provided evidence of the safety of cryotherapy for the skin. Despite the observed decrease in hydration, the mean water loss value (Robust Avg.) did not statistically significantly deteriorate, indicating that hydrolipid integrity was not compromised. Of note in the analysis of the results is the statistically significant variability of the Robust Spread parameter, which gradually decreased–in this study. This parameter reflects the standard deviation of the measurement over time. This trend may indicate functional adaptation of the epidermis and subsequent stabilization of thermoregulatory processes after a prior thermal shock. However, when interpreting this variability, the possibility of measurement error should be considered. In the case of Tewameter, including the Spread parameter reading, factors such as changes in the probe angle, air movement in the testing room, or the subject’s movement may influence the obtained values. Regardless of the interpretation of the dispersion parameter, the lack of increase in mean TEWL remains crucial. These observations correspond with existing literature reports indicating a lack of significant changes in transepidermal water loss in the forearm following cold exposure (Piotrowska et al., 2021). This confirms that the procedure used does not negatively affect the hydrolipid film, constituting a safe form of biological regeneration for the skin. Analyzing the lack of significant changes in TEWL values, it is worth considering the effect of exposure time on the adaptive mechanisms of the epidermis. Long-term winter swimming (two seasons) significantly remodels the barrier function of the epidermis, manifesting itself through its sealing, i.e., an improvement in the transepidermal water loss parameter (Polak-Bielawska et al., 2025).

Unlike vascular reactions and changes in hydration, no statistically significant differences were observed in any of the measured mechanical parameters after the treatment series. This analysis included assessment of collagen strength, firmness, elasticity, fatigue,–and viscoelasticity. The stability of the R0, R2, and R5 indices demonstrates that cyclic exposure to extremely low temperatures does not adversely affect collagen and elastin fibers. The constant level of the analyzed values ​​demonstrates that the skin did not change its elasticity. The lack of significant improvement can be explained by the specificity of the study group. The participants were young and healthy individuals, in whom physiological skin elasticity is naturally maintained at a very high level. The stabilization of results may also be due to the fact that the series of ten treatments was insufficient to induce measurable changes in the skin’s mechanical structure. An important conclusion is that despite the decrease in epidermal hydration, the biomechanical stability of the dermis was fully preserved. The conclusions drawn from the review of available literature indicate that the impact of low temperatures does not necessarily entail immediate structural remodeling, but rather focuses on stimulating metabolism in the dermis. According to the literature, significant effects are typically observed in cases such as advanced aging processes or weakened supporting fibers. The authors suggest that in the age group selected for this study, cryotherapy serves a conditioning and preventative function. It stimulates tissue nourishment without disrupting its natural mechanical homeostasis or stiffening elastin fibers, which is crucial for maintaining youthful skin structure (Dzidek and Piotrowska, 2022).

When analyzing the obtained results in the context of improving wellness procedures, it is worth considering the influence of kinesiotherapy as a factor increasing the potential effect of low temperatures. In this study, the procedure was limited to exposure to cold, followed by measurements immediately after exiting the chamber to immediately capture the effect. However, the literature indicates that WBC produces the best metabolic and vascular results when combined directly with physical activity (Piotrowska et al., 2021). The lack of a physical component may have weakened the potential therapeutic effect observed in our own studies (Ciosek et al., 2016). Consistent conclusions also emerge from analyses suggesting that the combination of physical and motor stimuli is crucial for the effectiveness of the therapy, especially in functional and analgesic aspects (Pietrzak et al., 2017). Regarding skin parameters, increased blood flow induced by exercise could accelerate the physiological recovery of tissue temperature and increase the transport of water and lipids to the stratum corneum, potentially offsetting the loss of hydration. This suggests that kinesiotherapy should be included in future studies on the effects of WBC on the skin. In the proposed combined model (cryotherapy combined with kinesiotherapy/training), the muscle pump would be activated during exercise, leading to an even stronger and more sustained vascular response.

When interpreting the above data, it is important to consider the methodological limitations of this study: the small sample size and very specific sample characteristics (young, healthy women without chronic diseases or skin problems); the lack of monitoring of diet, fluid intake, and lifestyle; and the lack of a separate control group. Despite these factors, which could have influenced the assessment of the effects, the collected research material indicates that WBC can influence biophysical parameters of the skin. This represents a valuable addition to existing knowledge and a starting point for further research on the optimization of biological regeneration treatments. Future studies should consider extending the follow-up period and increasing the number of participants (including–a control group), which would allow for a more accurate and comprehensive assessment of the effectiveness of WBC. The sample size in this study was not calculated and there is a risk of bias, so generalizability to larger or more diverse populations is limited. Furthermore, including individuals with various medical conditions could provide valuable data on the impact of WBC on different population groups. It would also be important to include a wider age range of study participants, allowing for an assessment of whether WBC effectiveness changes with age. Future studies would also benefit from a holistic approach, taking into account additional factors such as physical activity, diet, and combining pressotherapy with other therapeutic methods, such as kinesiotherapy or medical training. This approach could lead to more comprehensive and reliable results, confirming the effectiveness of this method in a wide range of aesthetic and therapeutic applications.

## 5 Conclusions

The results of a study examining the effect of a series of WBC treatments on selected skin characteristics in young, healthy individuals, despite the many limitations mentioned above, allow us to draw the following conclusions:

1. A series of WBC treatments induces a vascular response, manifested by increased skin redness (Erythema, Mexameter) and dynamic changes in surface temperature (Thermometer) in young, healthy women.
2. Despite the observed decrease in hydration (Corneometer), WBC treatments likely do not damage the hydrolipid barrier, as no increase in transepidermal water loss (TEWL Avg, Tewameter) was demonstrated in young, healthy women.
3. The effects of extremely low temperatures do not negatively affect the mechanical properties of skin: firmness, elasticity, and viscoelasticity (R0-R5, Cutometer) in young, healthy women.

Considering the conclusions from our study and the limitations discussed previously, further research is necessary to explain the complexity of these processes.

## Data Availability

The original contributions presented in the study are included in the article/supplementary material. Further inquiries can be directed to the corresponding author.
